# Two Novel Naphthalene Glucosides and an Anthraquinone Isolated from *Rumex dentatus* and Their Antiproliferation Activities in Four Cell Lines

**DOI:** 10.3390/molecules17010843

**Published:** 2012-01-17

**Authors:** Hui Zhang, Zengjun Guo, Nan Wu, Wenming Xu, Ling Han, Nan Li, Yanxia Han

**Affiliations:** Faculty of Pharmacy, School of Medicine, Xi'an Jiaotong University, No. 76 West Yanta Road, Xi'an 710061, China

**Keywords:** naphthalene, glucoside, anthraquinone, *Rumex dentatus*, antiproliferation

## Abstract

An ethyl acetate extract of the roots of *Rumex dentatus* L. was investigated. Three compounds were identified by their spectroscopic data as chrysophanol (**1**), 6-methyl-7-acetyl-1, 8-dihydroxy-3-methoxy naphthalene-1-*O*-β-D(L)-glucoside (**2**) and 6-methyl-7-acetyl-1, 8-dihydroxy naphthalene-1-*O*-β-D(L)-glucoside (**3**) were found in the plant for the first time. Compounds **2** and **3** are novel compounds. Their antiproliferation activities were tested by the MTT assay in four cell lines (breast cancer MCF-7, gastric cancer 7901, melanoma A375 and oophoroma SKOV-3).

## 1. Introduction

*Rumex*, the second genera among the *Polygonaceae*, comprises about 150 species widely distributed around the World. In China, it is represented by 26 species. *Rumex dentatus* is one of them, which could be found almost everywhere in China [1]. This traditional herb has been used as a medicine for many kinds of bacterial and fungal infection diseases, such as dysentery, enteritis, acariasis and eczema [2]. Since *Rumex* is in the same family with *Rheum*, their chemical compositions have some common features, such as the presence of chrysophanol, emodin, aloe-emodin and physcion [3,4,5,6,7,8]. Besides anthraquinones, other main chemical constituents in *Rumex* are flavonoids [9,10,11,12,13], diphenylethenes [10,14] and naphthalenes [15,16]. Research on the chemical contents of *Rumex dentatus* is rare. At this point, the chemical constituents from its root extract were scanned in our lab. Five compounds were obtained and three of them were identified by their physicochemical properties and spectroscopic analysis. Their antiproliferation activities were then tested with the MTT assay in four cell lines, including breast cancer MCF-7, gastric cancer 7901, melanoma A375 and oophoroma SKOV-3 and IC_50_ values in each cell line were calculated.

## 2. Results and Discussion

**Figure 1:**
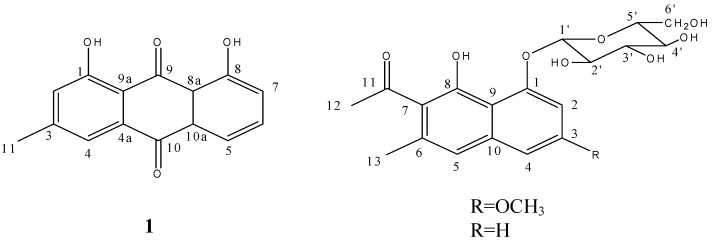
Structures of compounds **1**–**3**.

The Feigl reaction of compound **1** was positive, which indicated this compound might be a quinone. The magnesium acetate reaction showed an orange color, indicating the presence of a β-OH or an α-OH located on the benzene ring or the two –OH that were not on the same ring. The molecular formula C_15_H_10_O_4_ was assigned from its HRFABMS (*m/z* 255.3316 [M+H]^+^, calcd. 255.3399) and ^1^H, ^13^C-NMR data (Table 1). By comparing the NMR data with reported ones [17], this compound was identified as 3-methyl-1, 8-dihydroxy anthraquinone, that is to say, chrysophanol (Figure 1).

**Table 1:** ^1^H and ^13^C-NMR data for chrysophanol (compound **1**) (500 and 125 MHz, CDCl_3_, *J* in Hz and δ in ppm).

No.	δ_H_	δ_C_
1	/	162.7
2	7.11 (1H, d, *J* 1)	124.5
3	/	149.3
4	7.66 (1H, d, *J* 1)	121.4
5	7.83 (1H, dd, *J* 8.5, 1)	119.9
6	7.67 (1H, t, *J* 8.5)	136.9
7	7.30 (1H, dd, *J* 8.5, 1)	124.6
8	/	162.4
9	/	192.6
10	/	182.0
11	2.47 (3H, s)	22.2
4a	/	115.9
8a	/	115.5
9a	/	108.2
10a	/	135.7
1-OH	12.03	/
8-OH	12.13	/

Compound **2** gave a positive Molish reaction, which suggested this compound might be a glucoside. The molecular formula was assigned as C_20_H_25_O_9_ from its HRFABMS (*m/z* 410.5401 [M+H]^+^, calcd. 410.5410) and ^1^H, ^13^C-NMR data. There are two methyl signals at δ 19.92 and δ 32.64, and a methoxyl signal at δ 55.80 in the ^13^C-NMR. Considering the DEPT 135 spectrum evidence, signals at δ 61.25 (CH_2_), δ 70.43 (CH), δ 73.95 (CH), δ 76.77 (CH) and δ 78.28 (CH) should belong to a sugar. Three methenyl signals were observed at δ 101.75, δ 103.20 and δ 103.55. As one of them should be the terminal carbon of the sugar, the other two plus eight signals from δ 109.15 to δ 158.80 indicated the skeleton of this compound should be a naphthalene.

**Figure 2:**
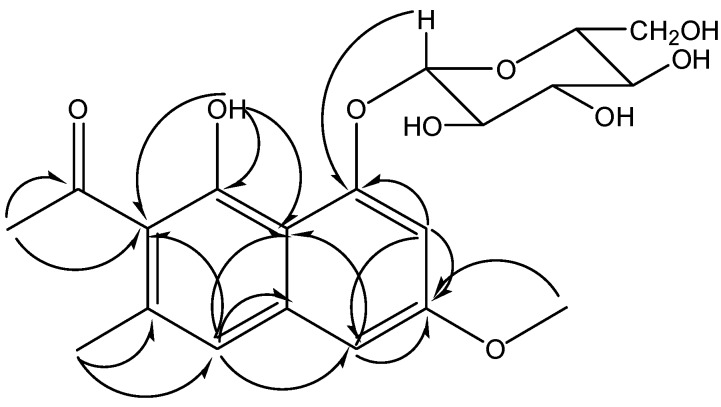
The key HMBC correlations of compound **2**.

At the lowest field, there is a carbonyl signal at δ 204.86. Two methyl proton signals were also observed in the ^1^H-NMR at δ 2.23 (s, 3H) and δ 2.51 (s, 3H) and a methoxyl proton signal was found at δ 3.84 (s, 3H). Six methenyl proton signals from δ 3.19 to δ 3.84 should belong to the sugar. The signal at δ 5.06 (d, *J* = 7.5 Hz, 1H) should be the terminal proton of the sugar. Aromatic proton signals were grouped according to their coupling and splitting. Signals at δ 6.91 (d, *J* = 2.5 Hz, 1H) and δ 7.00 (d, *J* = 2.5 Hz, 1H) were divided into group one and the signal at δ 7.09 (s, 1H) was in group two. The coupling constant of group one suggested these two protons should be in a *meta*-position, and since group two has only one proton signal without splitting, it should located at the other benzene ring of the naphthalene. Emulsin hydrolysis and TLC detection indicated the sugar should be a D-sugar, but the absolute configuration of the carbohydrate couldn’t be determined since no optical rotation data could be provided. Acid hydrolysis and gas chromatography (GC) analysis showed the presence of glucose. With consideration of the chemical shifts of the carbon and proton, as well as the coupling constant of the terminal proton, the sugar was conjectured to be β-D-glucose. The correlation from the terminal proton to the carbon signal at δ 103.20 in HSQC spectrum and the carbon signal at δ 155.87 in HMBC spectrum suggested which was the terminal carbon and where the glucose was located. The glycosidation position was labeled as position 1, then the correlations between C-1 and H-2 (δ 7.00), H-2 and C-3 (δ 158.80), C-3 and H-4 (δ 6.91) in HMBC (see Figure 2) clarified the sequence of the A ring. The correlation from the methoxyl proton (δ 3.84) to C-3 indicated the location of the methoxyl. The ^1^H-NMR analysis showed an uncoupled aromatic proton (δ 7.09) on ring B, and the correlation from it to C-4 in HMBC told us this proton should be labeled as H-5, so the carbon at δ 119.26 which correlated with H-5 in HSQC is C-5. The methyl proton at δ 2.23 which correlated with C-5 in HMBC was determined to be located at C-6 (δ 134.13). The correlation from this methyl to the carbon at δ 123.72 suggested the chemical shift of C-7. Correlations from the other methyl group (δ 2.51) to C-7 and the carbonyl carbon (δ 204.86) indicated the substitution of an acetyl at C-7. Putting all the above atoms together, there’s a –OH missing when compared with the molecular formula. So the only position left (δ 151.52) should connect with a hydroxyl. In summary, compound **2** was identified as 6-methyl-7-acetyl-1,8-dihydroxy-3-methoxynaphthalene-1-*O*-β-D(L)-glucoside (Figure 1). All the proton and carbon signals assignments was established with HSQC, ^1^H-^1^H COSY and HMBC (Table 2).

**Table 2:** ^1^H and ^13^C-NMR data for compounds **2** and **3** (500 and 125 MHz, DMSO-*d*_6_, *J* in Hz and δ in ppm).

No	2			3	
	δ_H_	δ_C_		δ_H_	δ_C_
1	/	155.9		/	154.8
2	7.00 (1H, d, *J* 2.5)	103.5		7.32 (1H, d, *J* 8)	111.2
3	/	158.8		7.41 (1H, t, *J* 8)	128.0
4	6.91 (1H, d, *J* 2.5)	101.7		7.48 (1H, d, *J* 8)	122.8
5	7.09 (1H, s)	119.3		7.23 (1H, s)	120.0
6	/	134.1		/	133.4
7	/	123.7		/	125.8
8	/	151.5		/	150.8
9	/	109.1		/	113.7
10	/	137.3		/	136.3
11	/	204.9		/	205.2
12	2.51 (3H, s)	32.6		2.53 (3H, s)	32.5
13	2.23 (3H, s)	19.9		2.26 (3H, s)	19.7
1'	5.06 (1H, d, *J* 7.5)	103.2		5.07 (1H, d, *J* 7.5)	103.2
2'	3.36 (1H, overlapping)	73.9		3.38 (1H, overlapping)	73.9
3'	3.34 (1H, overlapping)	76.8		3.37 (1H, overlapping)	76.8
4'	3.19 (1H, m)	70.4		3.21 (1H, dd, *J* 8.5, 5.5)	70.3
5'	3.47 (1H, overlapping)	78.3		3.44 (1H, t, *J* 9.5, 6)	78.3
6'	3.50 (1H, overlapping )	61.2		3.51 (1H, ddd, *J* 12, 6, 6)	61.2
	3.75 (1H, dd, *J* 11, 5.5)			3.77 (1H, dd, *J* 10.5, 5.5)	
8-OH	9.50 (1H, s)	/		9.60 (1H, s)	/
3-OMe	3.84 (3H, s)	55.8		/	/

Compound **3** also showed a positive Molish reaction. The molecular formula was assigned as C_19_H_23_O_8_ from its HRFABMS (*m/z* 379.5006 [M+H]^+^, calc. for 379.5008) and ^1^H-, ^13^C-NMR data, which suggested this compound might have a similar skeleton as compound **2**, except for a methoxyl group. Comparing the NMR spectra of these two compounds, the disappearances of the methoxyl carbon signal at δ 55.8 and the methoxyl proton signal at δ 3.84 also supported this deduction. Therefore, compound **3** was identified as 6-methyl-7-acetyl-1,8-dihydroxynaphthalene-1-*O*-β-D-glucoside (Figure 1). The chemical shifts of all carbon atoms in these two compounds were compared to see the substituent chemical shift changing rules. As the chemical shifts of glucose and B ring are almost matched, the signal of C-3 shifted down-field from δ 128.0 to δ 158.8 and signals of C-2 and C-4 shifted up-field from δ 111.2 to δ 103.5 and from δ 122.8 to δ 101.7 due to the substituent of methoxyl at C-3. In addition as the *para*-position of C-3, the signal of C-9 shifted up-field from δ 113.7 to δ 109.1. With all this proof, the previous hypothesis was confirmed. The assignment of all the proton and carbon signals was established with HSQC, ^1^H-^1^H COSY and HMBC (Table 2).

The compounds above were further evaluated for their antiproliferative activity using four cell lines including MCF-7 breast cancer cell line, gastric cancer 7901 cells, melanoma A375 cells and oophoroma SKOV-3 cells. Their antiproliferation activities were represented with IC_50_ values (Table 3). Chrysophanol (**1**), was more active than the other two, especially in the oophoroma SKOV-3 cell line, where the IC_50_ value was 5.62 μM. Compounds **2** and **3** showed no effects on the gastric cancer 7901 cells. The methoxyl group at C-3 in **2** seems to have a key influences of the antiproliferation activity since most of the IC_50_ values of **2** were lower than those of **3**.

**Table 3:** IC_50_ values (μM) of compound **1–3** in four cell lines.

Compound	MCF-7	7901	A375	SKOV-3
	breast cancer	gastric cancer	melanoma	oophoroma
**1**	20.4 ± 7.8	513 ± 265	83.1 ± 35.1	5.62 ± 1.58
**2**	269 ± 133	-	186 ± 57	40.7 ± 23.1
**3**	1580 ± 1860	-	275 ± 143	174 ± 114

## 3. Experimental

### 3.1. General

Melting points were determined on a MP-J3 microscope apparatus. UV were obtained on a SP-2102UV spectrophotometer. IR spectra (KBr) were recorded on a Jasco FTIR-4100. The NMR spectra were recorded on a Bruker Ultrashield Plus spectrometer (500 MHz for ^1^H-NMR and 125 MHz for ^13^C-NMR) with TMS as internal standard. The HR-ESI-MS were obtained with a Bruker APEX III spectrometer. GC were performed on a Shimadzu GC-QP2010. The OD values in MTT assay were measured by a POLARstar + OPTIMA Plate Reader (BMG Labtechnologies, Ortenberg, Germany). The purity of compounds were checked on a Waters 600 (Waters, Milford, MA, USA) HPLC system equipped with an Intersel C_18_ (5 μm, 4.6 × 250 mm) column. Column chromatography was performed with silica gel (200–300 mesh, Qingdao Haiyang Chemical Group Co. Ltd, Qingdao, China). TLC were detected on silica gel 60 F254 (Merck, Darmstadt, Germany) by spraying with 10% ethanolic H_2_SO_4_ reagent followed by heating.

### 3.2. Plant Material

*Rumex dentatus* L. roots were collected from the test herb field of Xi’an Jiaotong University, Xi’an, China, in December 2010. The plant was identified by Professor Junxian Wang at Xi’an Jiaotong University of Nature Products Chemistry and a voucher specimen has been deposited in Faculty of Pharmacy, School of Medicine, Xi’an Jiaotong University, Xi’an 710061, China.

### 3.3. Cell Culture

Cell lines were obtained from Shanghai Institute of Biochemistry and Cell Biology, Chinese Academy of Sciences (Shanghai, China) and routinely cultured in RPMI-1640 (breast cancer MCF-7, melanoma A375 and oophoroma SKOV-3) or DMEM (gastric cancer 7901) medium supplemented with 10% fetal bovine serum (FBS) and 1% penicillin/streptomycin in a humidified incubator at 37 °C with 5% CO_2_.

### 3.4. Extraction and Isolation

One kilogram of *Rumex dentatus* L. roots were collected and air-dried. The meshed herb was then refluxed with methanol (×8) for 3 h to obtain the crude extract. The residue was dissolved in H_2_O (1 L) and then extracted successively with petroleum ether (1 L × 3), chloroform (1 L × 3), ethyl acetate (1 L × 3) and *n*-butanol (1 L × 3). The ethyl acetate part (13 g) was then subjected to silica gel column chromatography and eluted with chloroform-methanol system in the ratios of 500:1, 400:1, 300:1, 200:1, 150:1, 100:1, 80:1, 60:1, 50:1, 30:1, 10:1, 5:1 and 1:1 to give 350 fractions. The crystals in fractions 26–38 and fractions 67–75 were recrystallized to yield compounds **1** (12 mg) and **2** (30 mg). Fractions 80–100 were further subjected to silica gel CC with chloroform-acetone (10:3). Subfractions 51–57 afforded compound **3** (56 mg).

Compound **1**, orange needles (methanol); mp 145–147 °С; [α]_D_^25^ +21.3 (c 0.55, acetone); UV λ_max_ (MeOH): 258.50, 289.00, 433.00; IR bands (KBr): 3451, 3024，2957, 1673, 1622, 1561, 1382, 1262, 1196, 1053, 989, 870 cm^−1^; ^1^H (500 MHz, CDCl_3_) and ^13^C-NMR (125 MHz, CDCl_3_) data: see Table 1; HRFABMS (positive ion mode) *m/z*: 255.3316 [M+H]^+^. Calc. for C_15_H_11_O_4_ 255.3399.

Compound **2**, white needles(acetone); mp 190–192 °С; [α]_D_^25^ +53.5 (c 0.50, MeOH); UV λ_max_ (MeOH): 225.78, 312.54; IR bands (KBr): 3431, 2948, 1725, 1576, 1567, 1557, 1409, 1383, 1266, 1180, 1054, 871 cm^−1^; ^1^H (500 MHz, DMSO-*d*_6_) and ^13^C-NMR (125 MHz, DMSO-*d*_6_) data: see Table 2; HRFABMS (positive ion mode) *m/z*: 410.5401 [M+H]^+^. Calc. for C_20_H_26_O_9_ 410.5410.

Compound **3**, white needles (acetone); mp 193–195 °С; [α]_D_^25^ +58.2 (c 0.55, MeOH); UV λ_max_ (MeOH): 224.60, 310.12; IR bands (KBr): 3478, 2928, 1742, 1557, 1526, 1384, 1256, 1184, 1054, 872 cm^−1^; ^1^H (500 MHz, DMSO-*d*_6_) and ^13^C-NMR (125 MHz, DMSO-*d*_6_) data: see Table 2; HRESIMS *m/z*: 379.5006 [M+H]^+^. Calc. for C_19_H_23_O_8_ 379.5008.

### 3.5. Hydrolysis

Compound **2** (2 mg) was dissolved in PBS (2 mL) and emulsin (20 μL, 80 u/μL) was added. Then the mixture was incubated in a shaker bath at 50 °С and 75 rpm for 5 h. The hydrolysis product was extracted with chloroform and concentrated. Then TLC was employed to determine if the indican was hydrolyzed to an aglycone.

Acid hydrolysis was performed by a reported method [18]. Briefly, compound **2** (10 mg) was dissolved in 12% HCl (5 mL) and heated at 90 °С for 2 h. The aglycone was extracted with chloroform, and the pH value of aqueous residue was adjusted to 7.0 with 12% NaOH. NaBH_4_ (40 mg) was added and the mixture was acidified with dilute CH_3_COOH. Excess boric acid was removed by distilling with methanol. Pyridine (1 mL) was added to dissolve the reduced sugar and acetic anhydride (1 mL) was then added. The whole mixture was heated at the room temperature for 12 h to afford the corresponding alditol acetates. The product was then analyzed by GC-QP2010 (Shimadzu), which was equipped with an FID and N_2_ was used as the carrier gas with flow speed at 1 mL/min. An HP-1 (30 m × 0.32 mm) capillary column was employed for the analysis and injector temperature was 280 °C. The temperature program was started at 180 °C, then increased to 280 °C at the speed of 10 °C/min after 10 min, and kept at 280 °C for 5 min. The standard sugar was treated the same way and analyzed under the same condition (D-glucose, t_R_, 16.8 min).

### 3.6. MTT Assay

To perform the antiproliferation assay, cells were plated in 96-well microtiter plates at the concentration of 1 × 10^6^/well in appropriate growth media (DMEM for gastric cancer and RPMI-1640 for others). The DMSO solution (1 mL) of test compounds were added 24 h later. Negative controls received normal growth media and the same amount of DMSO. Plates were incubated for 2 days after treatment, then 20 mL MTT solution (5 g/L) was added into each well and incubated for another 4 h. Supernatants were removed and formazan crystals were dissolved in 200 mL dimethylsulfoxide. A plate reader was used to measure the staining intensity of each well at 490 nm. The IC_50_ values were calculated with the SPSS software using five drug concentrations (10^4^–1 μM) and the assays were performed in triplicate.

## 4. Conclusions

Three compounds were obtained from an ethyl acetate extract of the root of *Rumex dentatus* L.and identified as chrysophanol (**1**), 6-methyl-7-acetyl-1,8-dihydroxy-3-methoxynaphthalene-1-*O*-β-D(L)-glucoside (**2**) and 6-methyl-7-acetyl-1,8-dihydroxynaphthalene-1-*O*-β-D(L)-glucoside (**3**) by their physicochemical properties and spectroscopic analysis. According to the references [3,4,5,6,7,8,9,10,11,12,13,14,15,16] and the SciFinder search system, these three compounds were found in the plant for the first time and compounds **2** and **3** were identified as novel compounds. The antiproliferation activities of compound **1** was better than those of the other two naphthalene compounds. Compound **2** showed higher activity than **3**, which might be due to the methoxyl group at C-3 in **2**. Both **2** and **3** showed no effects on the gastric cancer 7901 cell line, and even the activity of **1** on this cell line was weak. No reasonable explanations for these differences can be put forth at this time. 

## Supplementary Materials

Supplementary Materials can be accessed at: http://www.mdpi.com/1420-3049/17/1/843/s1.

## References

[1] Li A.R., Qian C.S., Chen H.Y. (1998). Polygonaceae. Flora of China.

[2] Song L.R., Hu L. (1999). *Rumex* *dentatus*. Traditional Chinese Herb.

[3] Varma P.N., Lobar D.R., Satsangi A.K. (1984). Phytochemical study of *Rumex acetosa* Linn. J. Indian Chem. Soc..

[4] Tamano M., Koketsu J. (1992). Isolation of hydroxyanthrones from the roots of *Rumex acetosa* Linn. Agric. Biol. Chem..

[5] Demirezer O.L., Kuruuzum A. (1997). Rapid and simple biological activity screening of some *Rumex* species evaluation of bioguided fractions of *Rscutatus* and pure compounds. Z. Naturforsch. C.

[6] Demirezer O.L., Ayse K., Isabelle B. (2001). The structures of antioxidant and cytotoxic agents from natural source anthraquinones and tannins from roots of *Rumex patientia*. Phytochemistry.

[7] Nishina A., Suzuki H. (1993). Naphthoquinone derivative of *Rumex japonicus* and *Rheum* as microbicide for foods. Jpn. Kokai Tokkyo Koho.

[8] Erturk S., Ozbas M., Imre S. (2001). Anthraquinone pigments from *Rumex cristatus*. Pharm. Turcica.

[9] Abd F.H., Gohar A., el-Dahmy S., Hubaishi A. (1994). Phytochemical investigation of *Rumex luminiastrum*. Acta Pharm. Hung..

[10] Kerem Z., Regev S.G., Flaishman M.A. (2003). Chemical constituents from *Rumex bucephalorus*. J. Nat. Prod..

[11] El-Fattah H.A., El-Dahmy S., Abdel-Aal M., Halim A.F., Abdel-Halim O.B. (1995). Phenolic compounds from *Rumex bucephalorus*. Sci. Pharm..

[12] Hasan A., Ahmed I., Khan M.A. (1997). A new anthraquinone glycoside from *Rumex chalepensis*. Fitoterapia.

[13] Zaghou M.G., El-Fattah H.A. (1999). Anthraquinones and flavonoids from *Rumex tingitanus* growing in Libya Zagazig. Pharm. Sci..

[14] Wang Z.Y., Zuo K.M., Kang Y.H. (2005). The research of chemical constituents in *Rumex gmelini* TurczII. Zhongcaoyao.

[15] Cetinkaya O., Siling Y., Cetinkaya S. (2002). The effects of *Rumex patientia* extract on rat liver and erythrocyte antioxidant enzyme system. Pharmazie.

[16] Kuruuzum A., Demirezer L.O., Bergere I. (2001). Two new chlorinated naphthalene glycosides from *Rumex patientia*. J. Nat. Prod..

[17] Jaki B., Heilmann J.O. (2000). New antibacterial metabolites from the Cyanobacterium *Nostoc commune*. J. Nat. Prod..

[18] Kuang H.X., Su Y., Yang B.Y., Xia Y.G., Wang Q.H., Wang Z.B., Yu Z.F. (2011). Three new cycloartenol triterpenoid saponins from the roots of *Cimicifuga simplex* wormsk. Molecules.

