# Supplementary material for: Two Novel Naphthalene Glucosides and an Anthraquinone Isolated from Rumex dentatus and Their Antiproliferation Activities in Four Cell Lines
Source: Molecules. 2012 Jan 17;17(1):843–50. doi: 10.3390/molecules17010843 (PMC6268050; doi:10.3390/molecules17010843)
Supplement: Supplementary file 1 [file molecules-17-00843-s001.doc]

**Supplementary**

**Figure 1.** ^1^H-NMR spectrum of compound **1**.


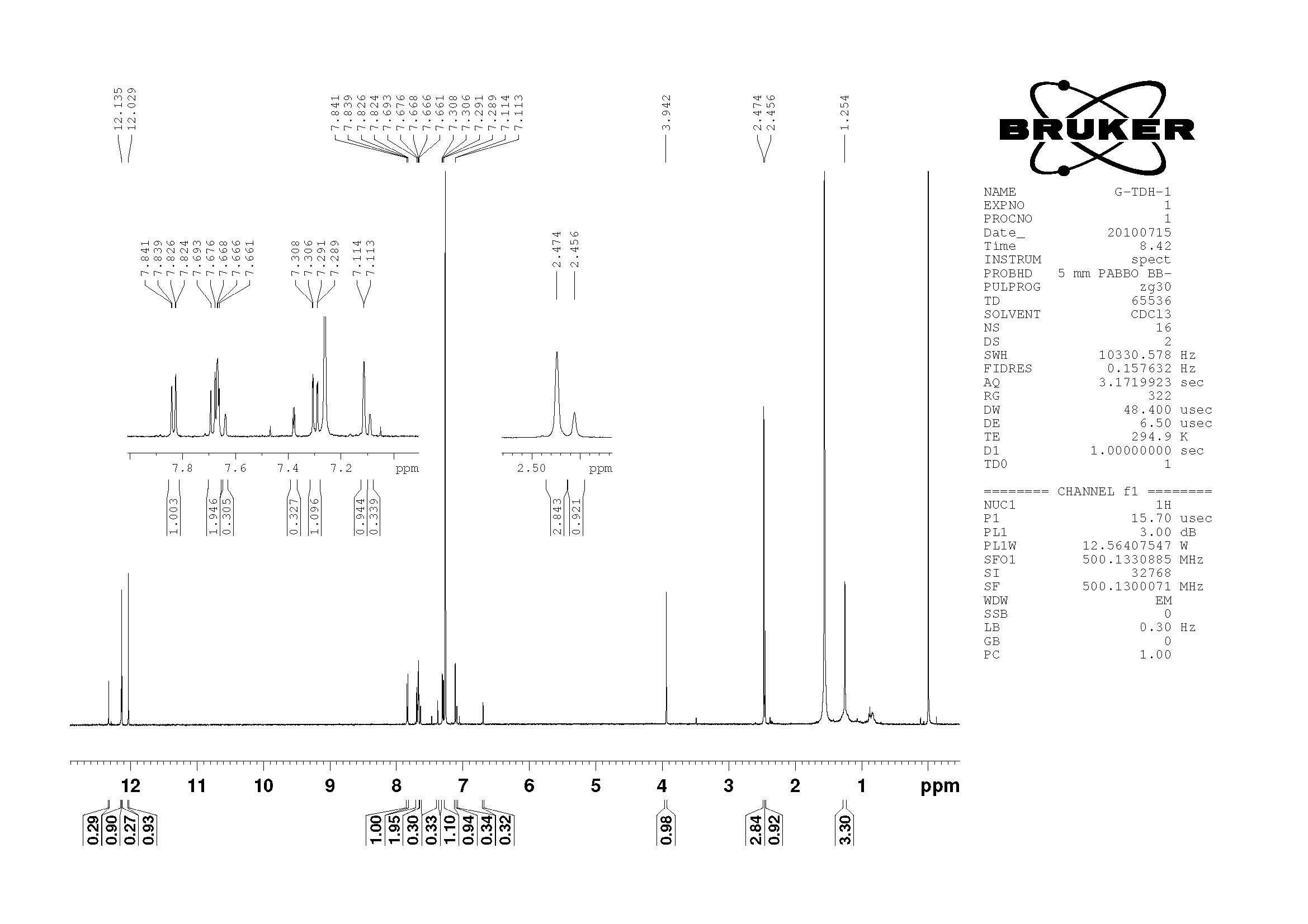


**Figure 2.** ^13^C-NMR spectrum of compound **1**.


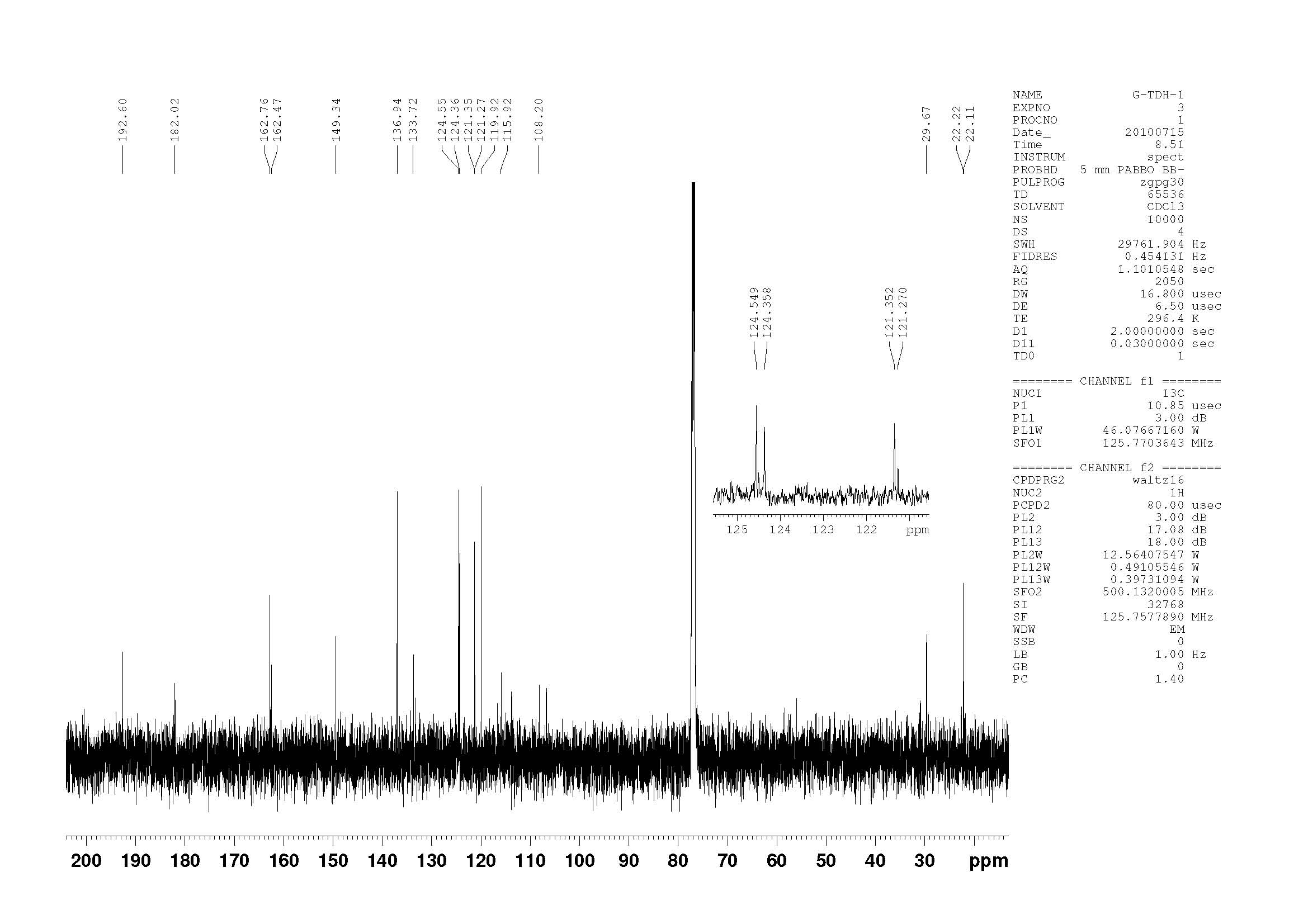


**Figure 3.** ^1^H-NMR spectrum of compound **2**.


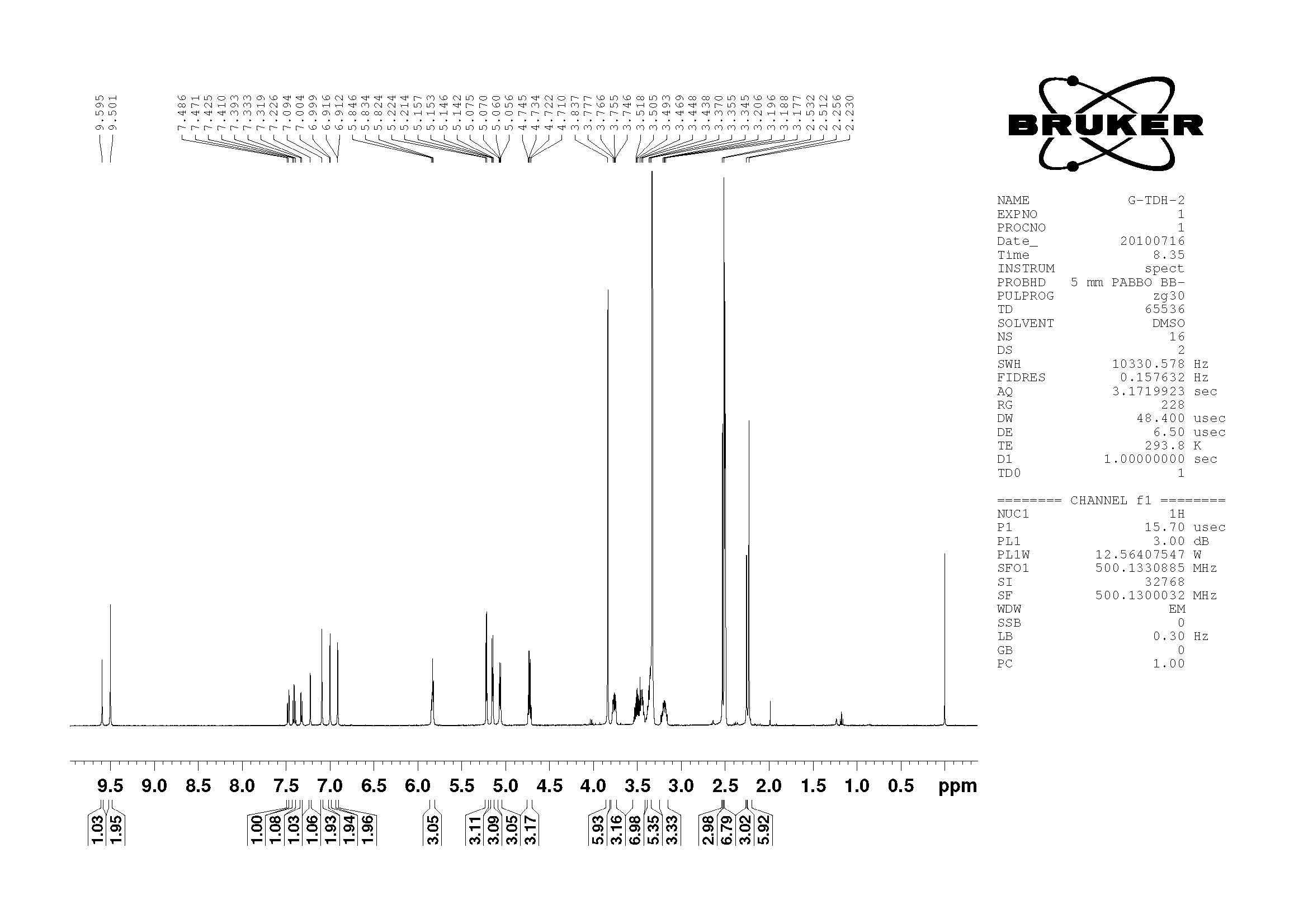


**Figure 4.** ^1^H-NMR spectrum of compound **2**.


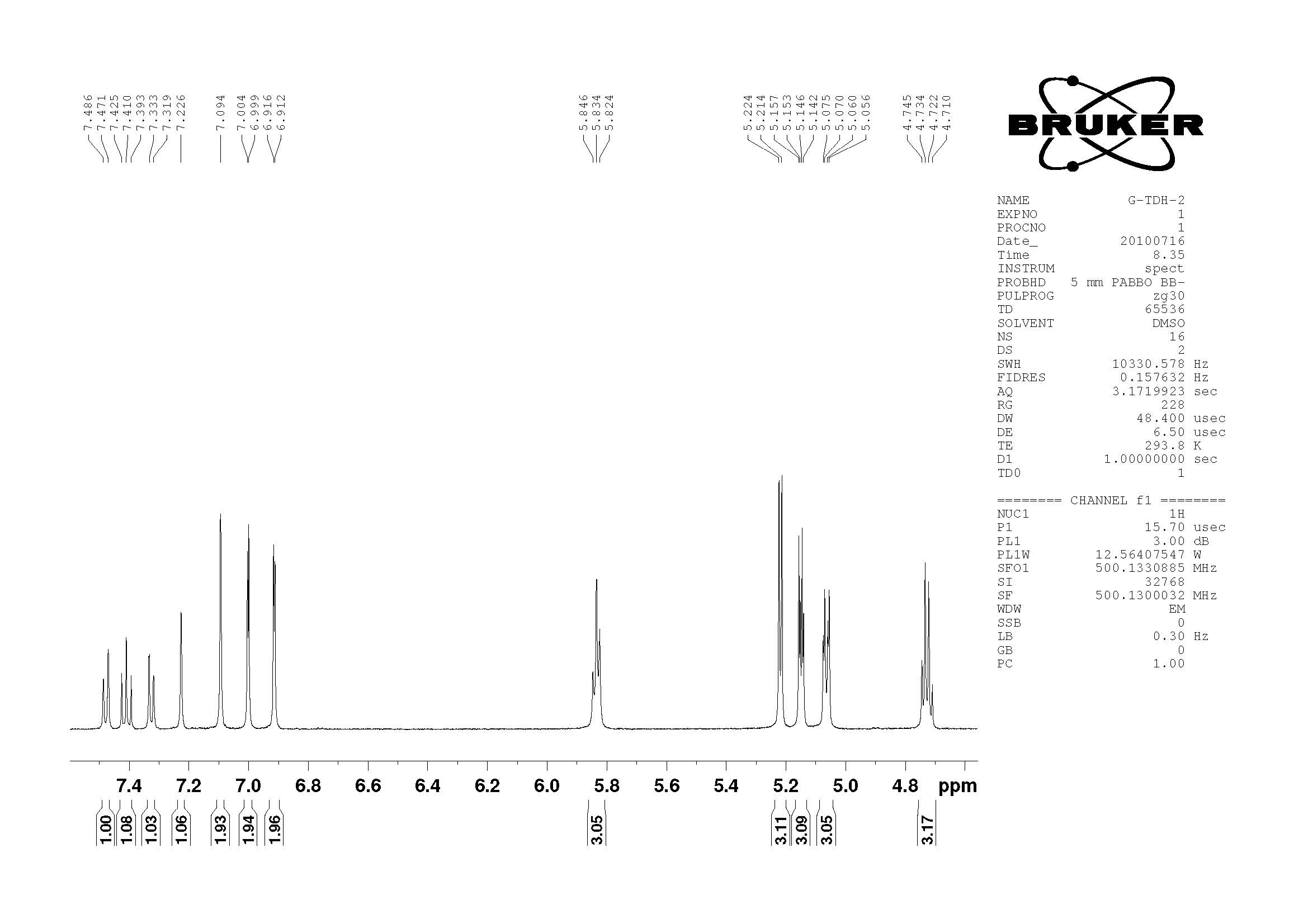


**Figure 5.** ^1^H-NMR spectrum of compound **2**.


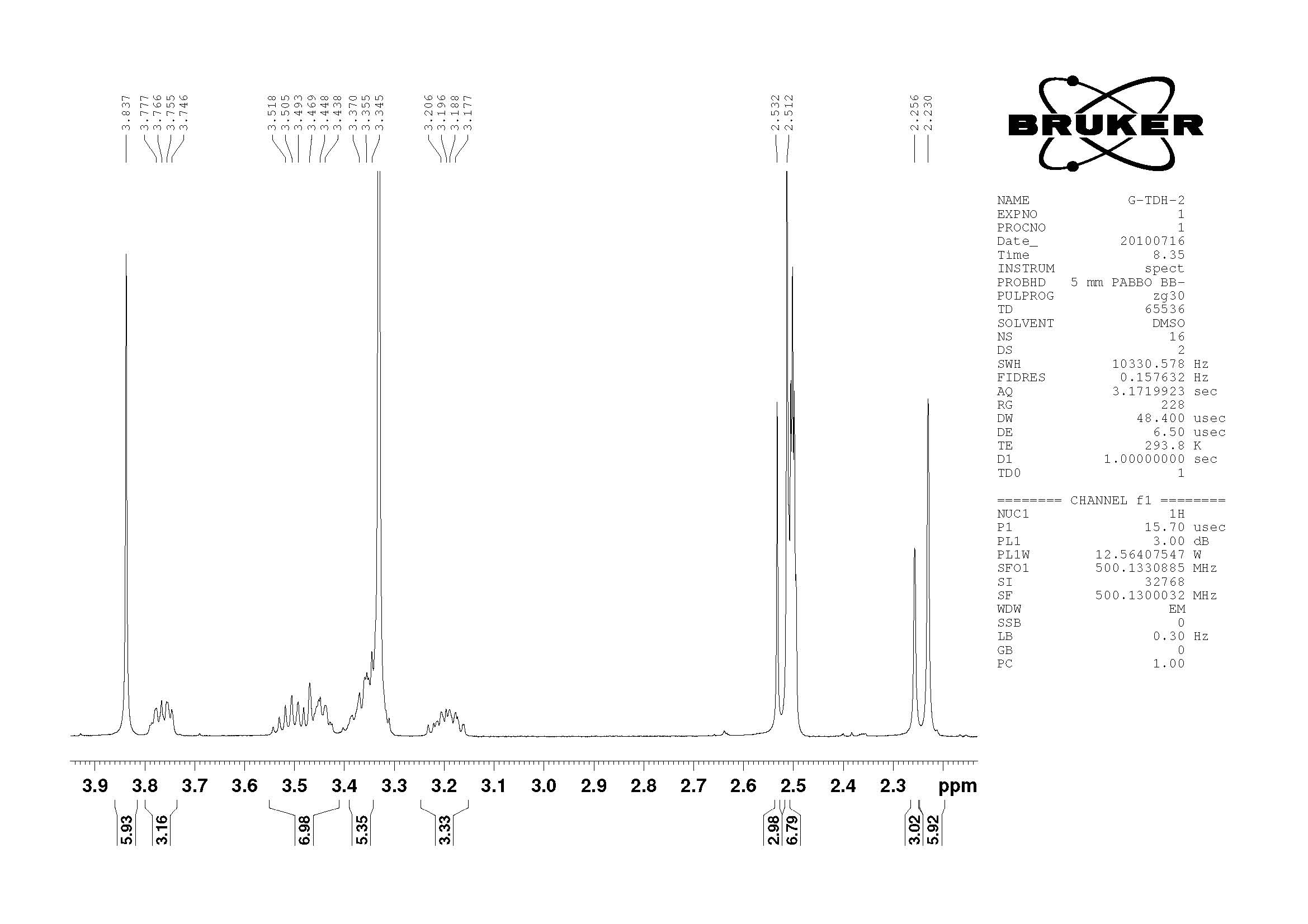


**Figure 6.** ^13^C-NMR spectrum of compound **2**.


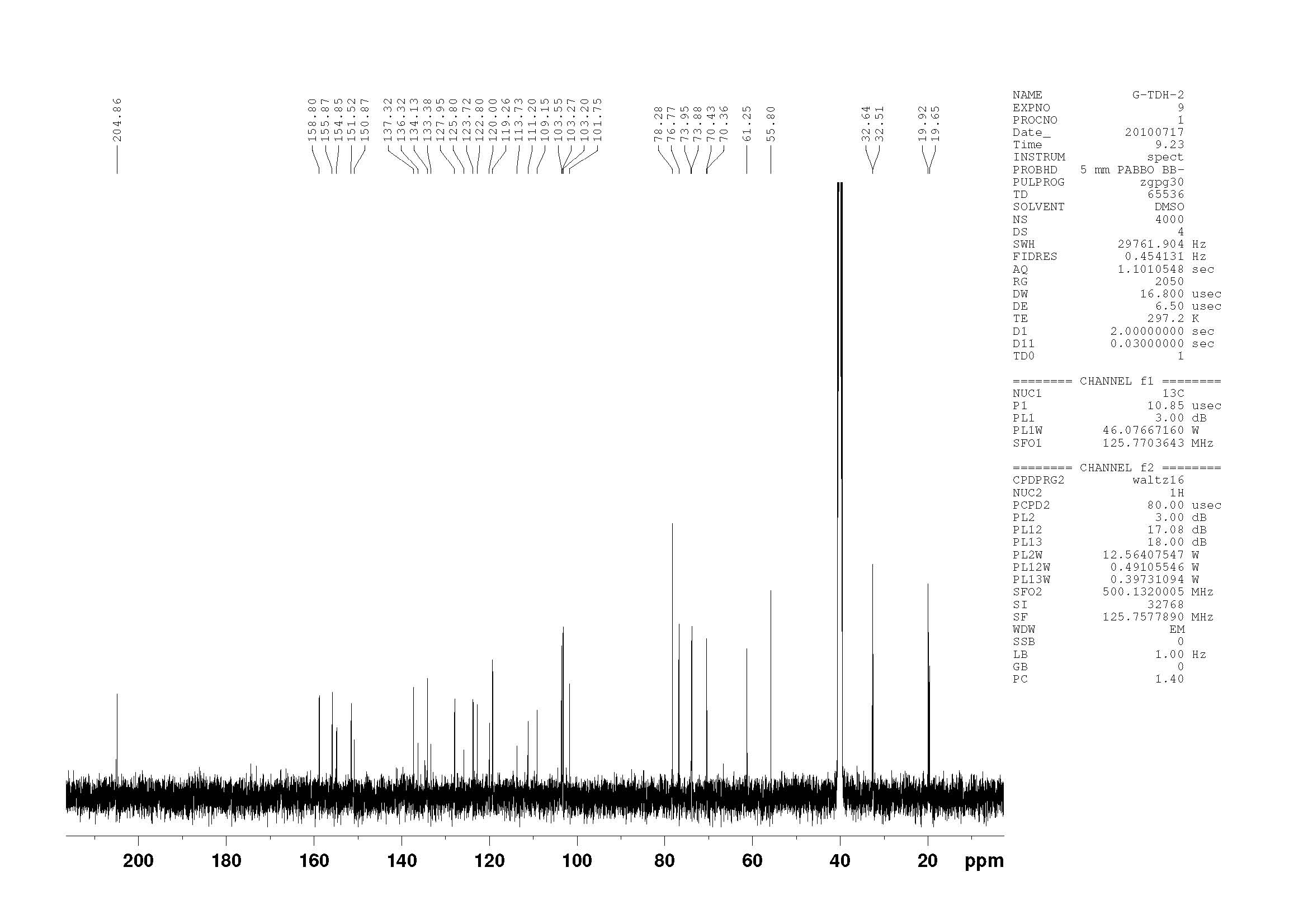


**Figure 7.** DEPT 135 spectrum of compound **2**.


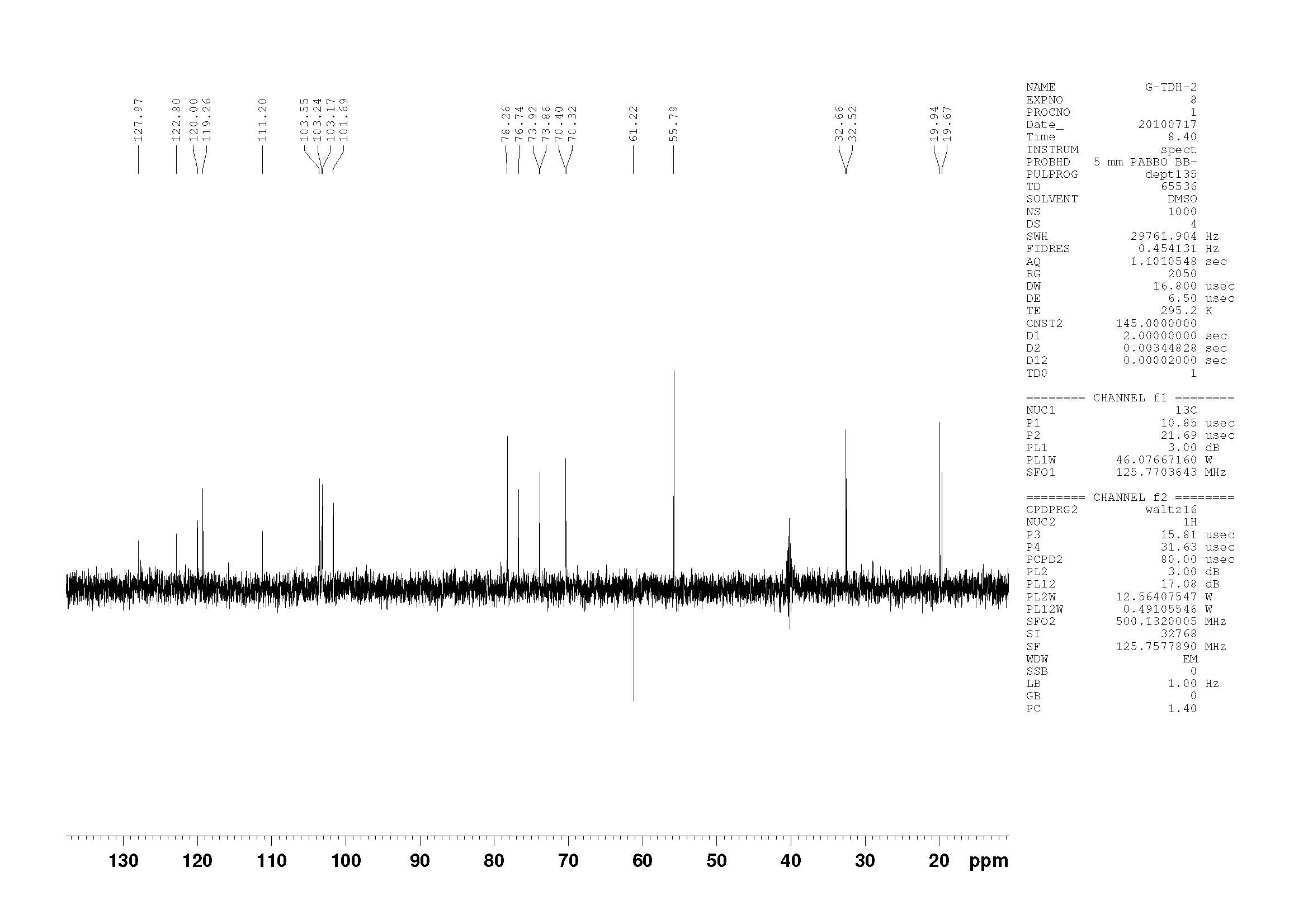


**Figure 8.** HSQC spectrum of compound **2**.


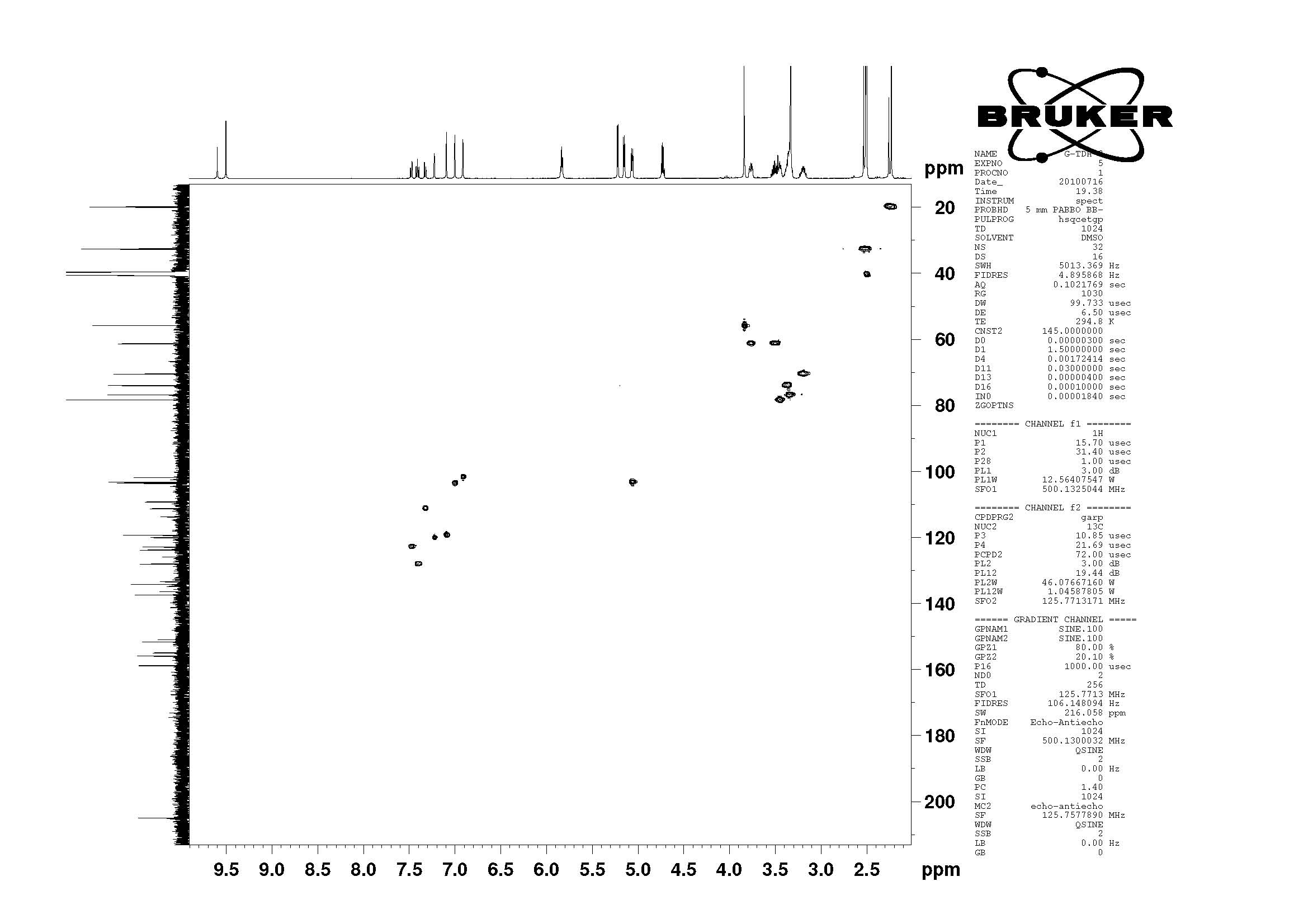


**Figure 9.** HSQC spectrum of compound **2**.


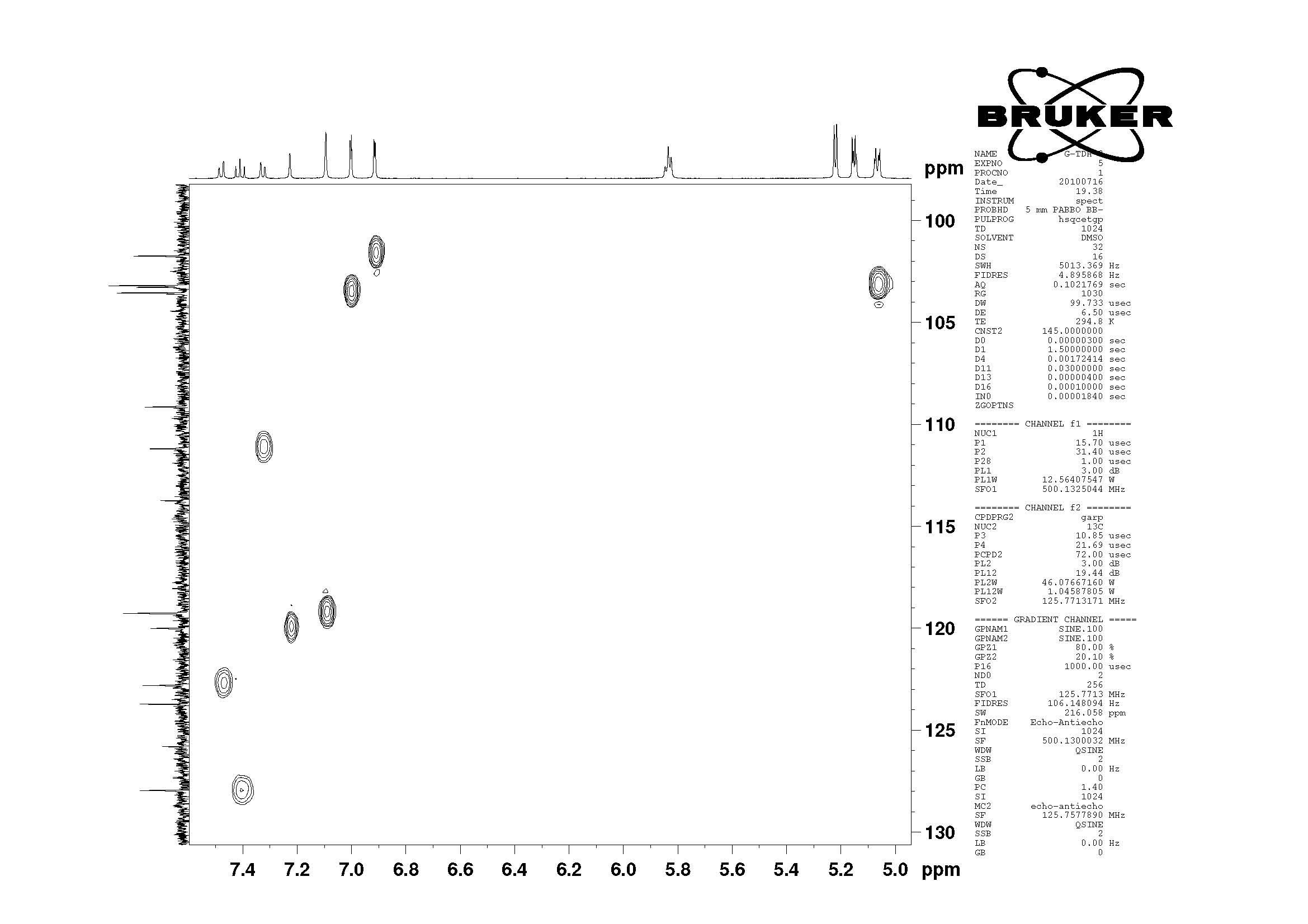


**Figure 10.** HMBC spectrum of compound **2**.


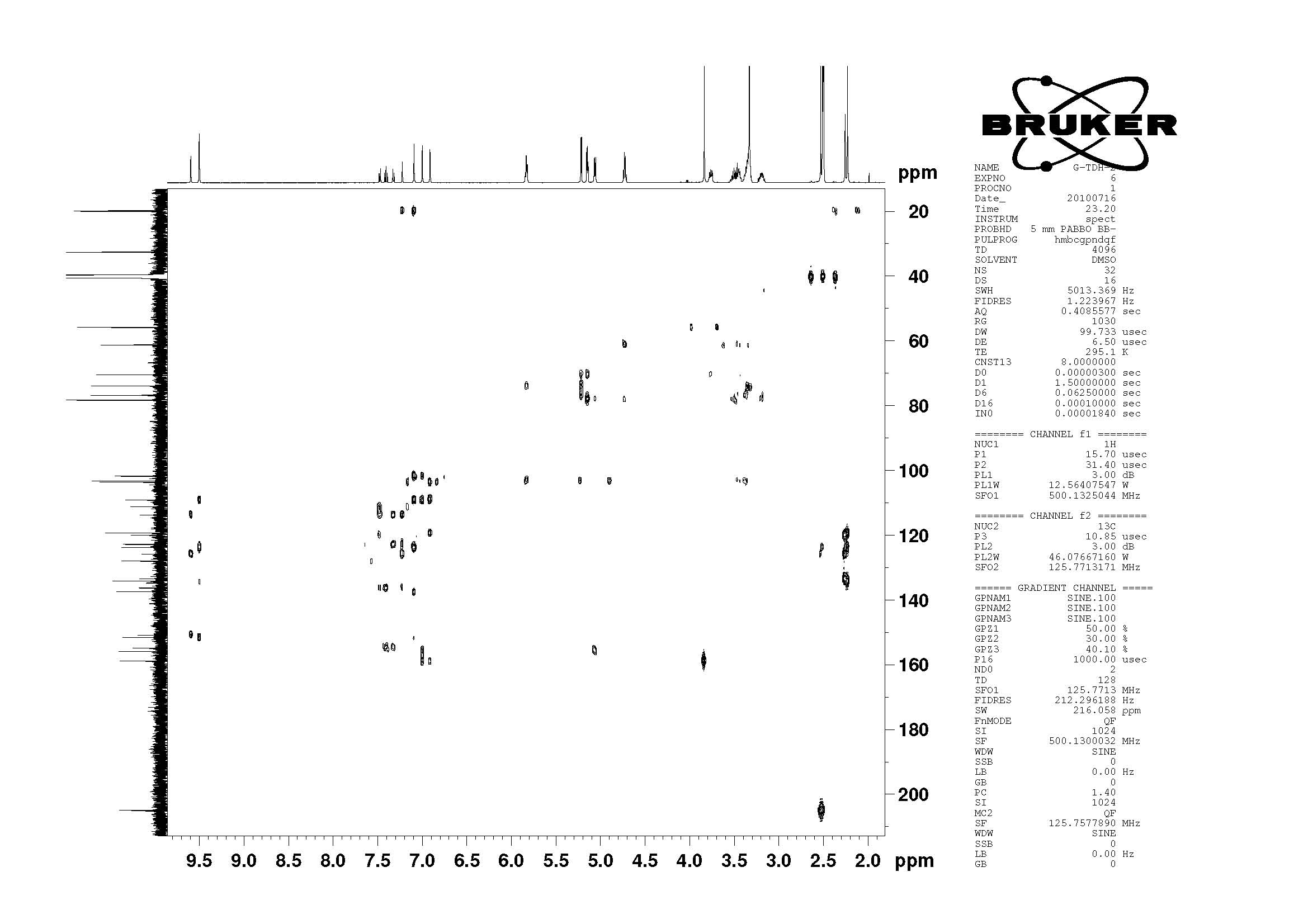


**Figure 11.** HMBC spectrum of compound **2**.


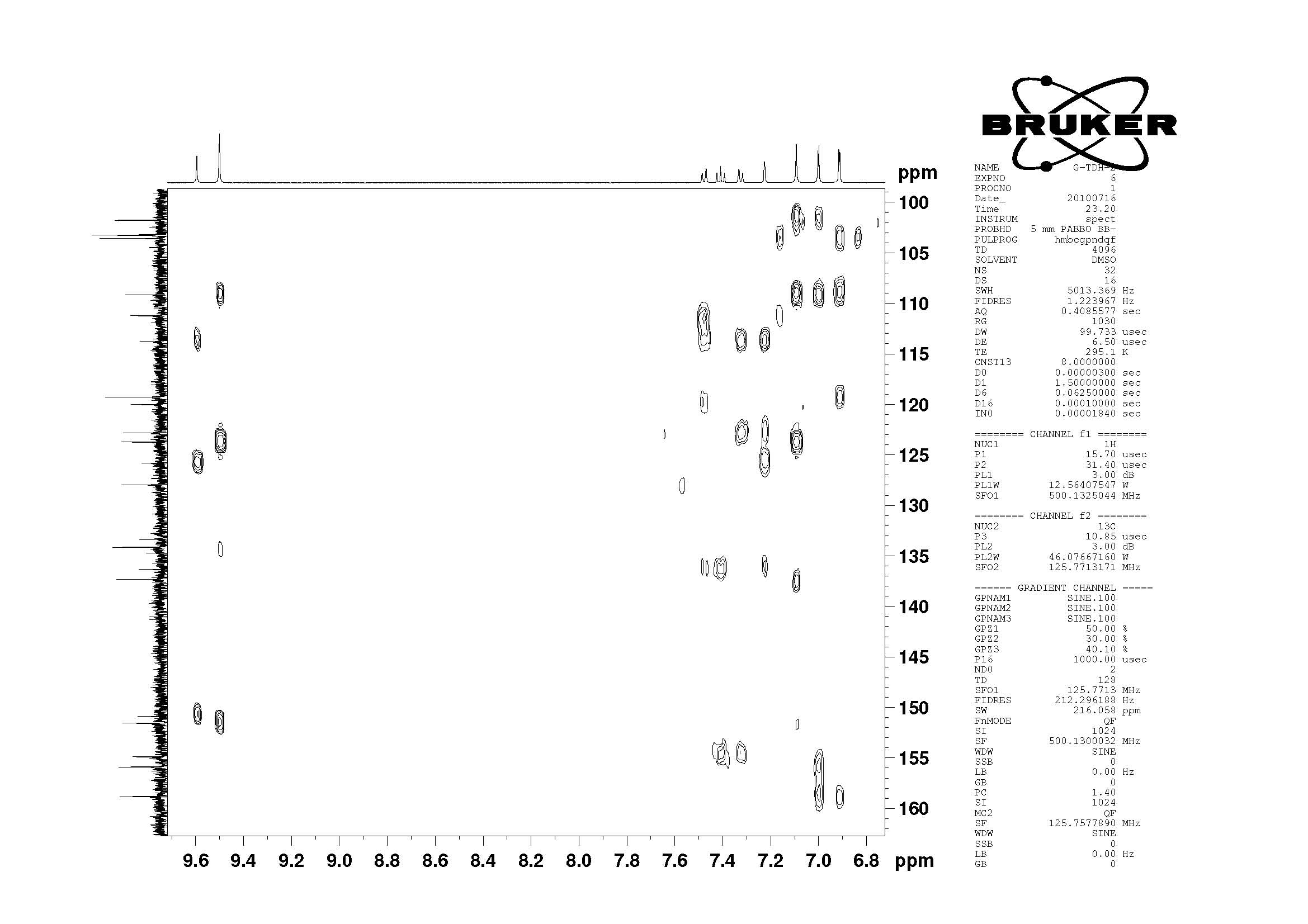


**Figure 12.** HMBC spectrum of compound **2**.


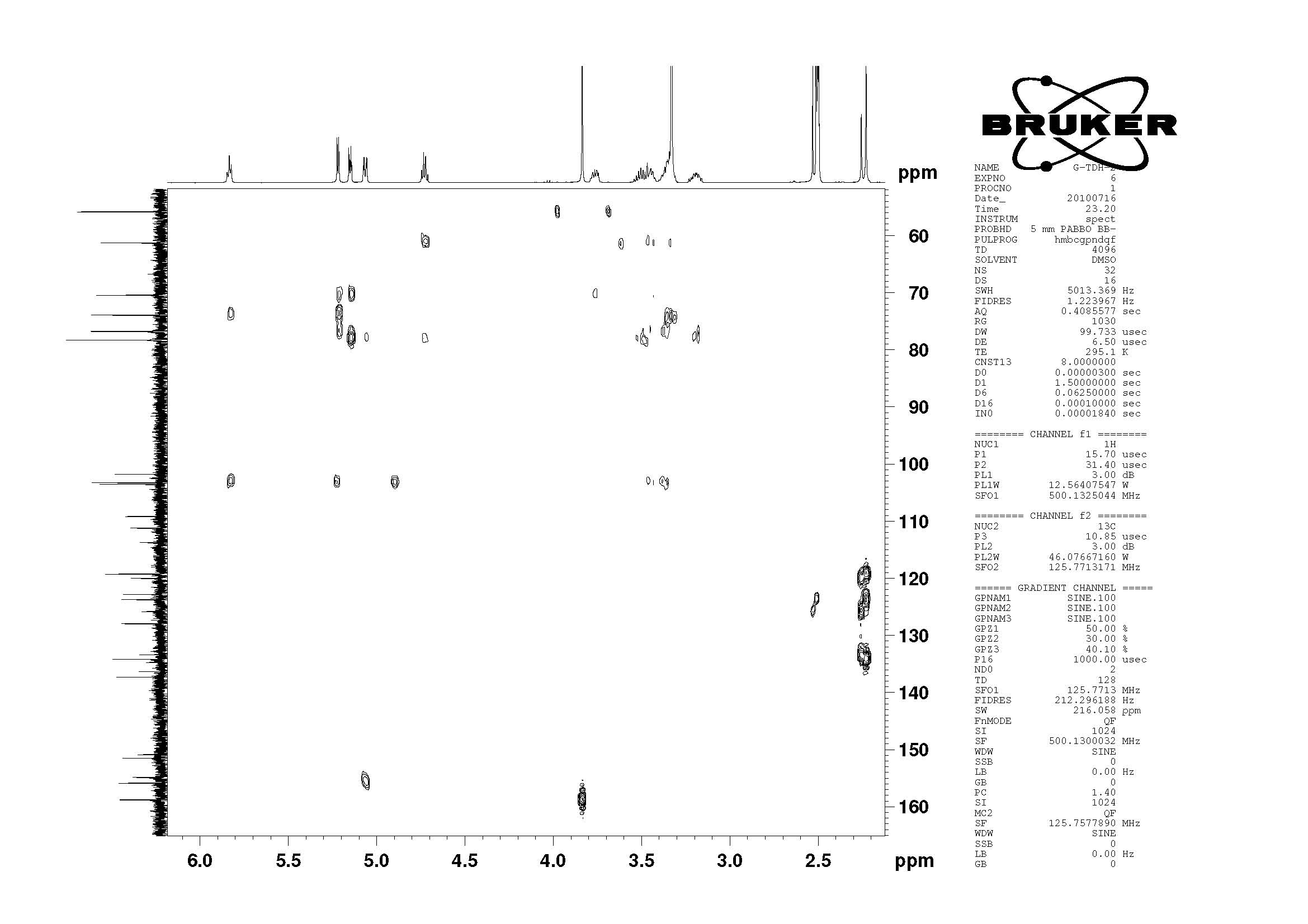


**Figure 13.** ^1^H-NMR spectrum of compound **3**.


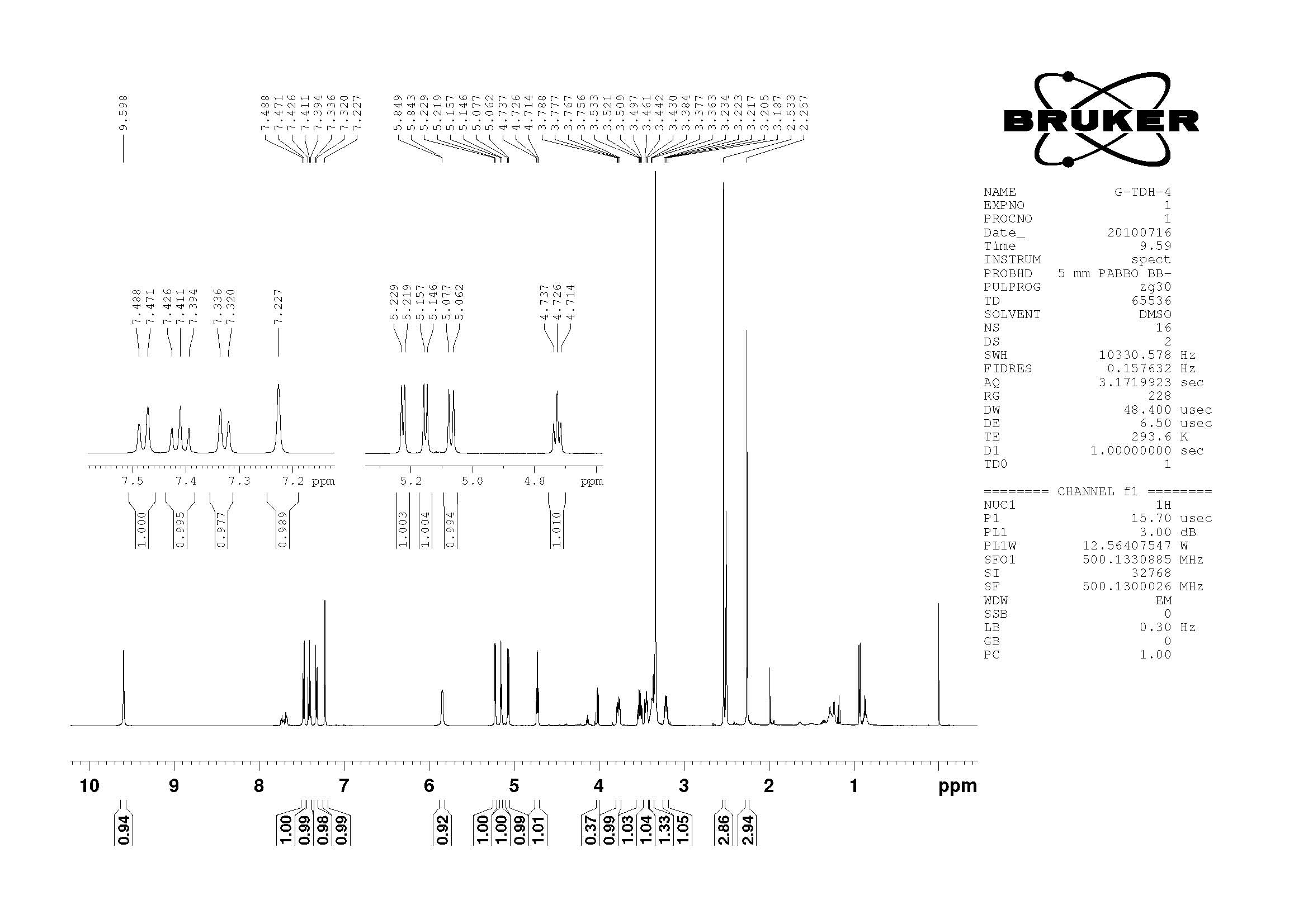


**Figure 14.** ^1^H-NMR spectrum of compound **3**.


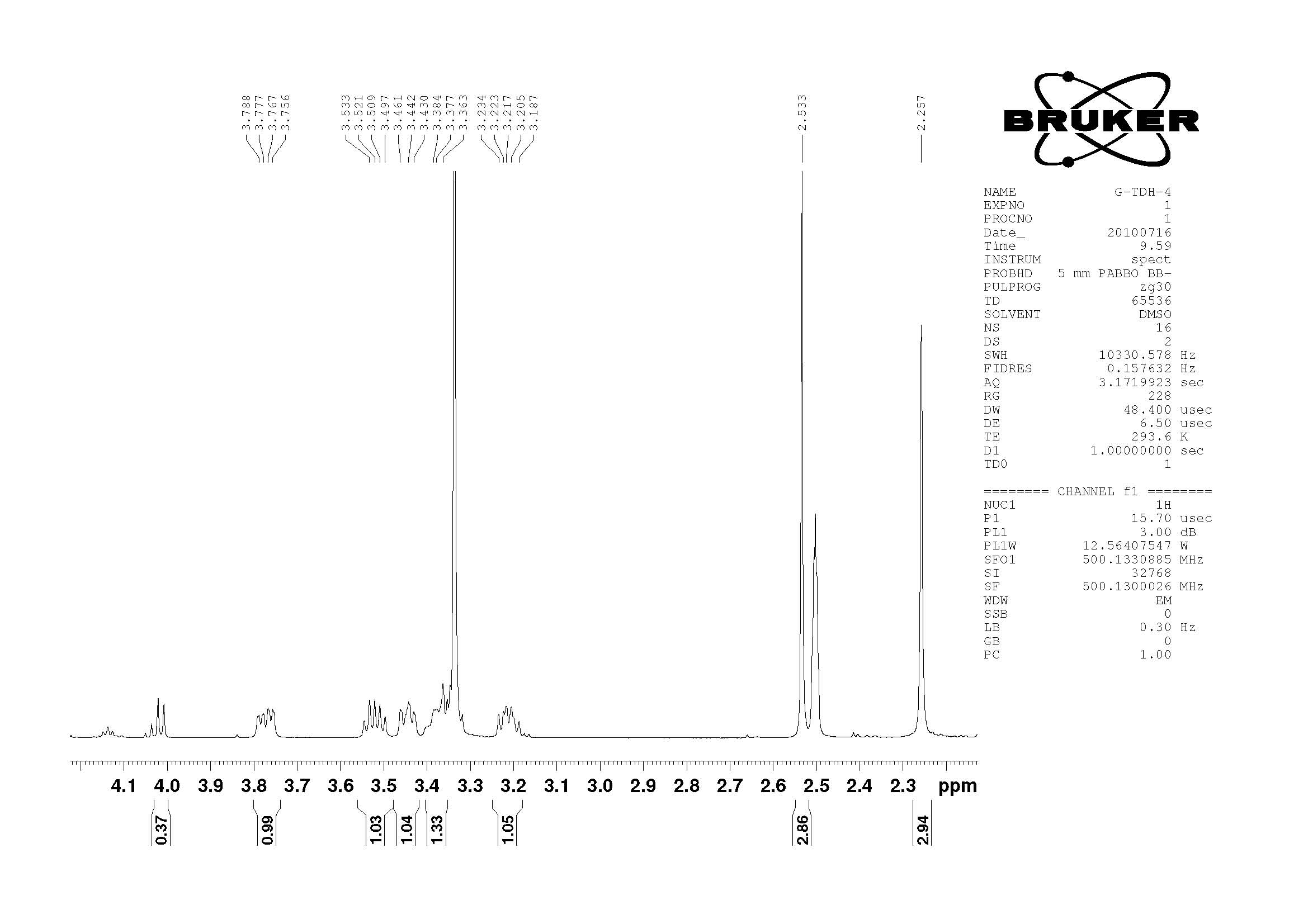


**Figure 15.** ^13^C-NMR spectrum of compound **3**.


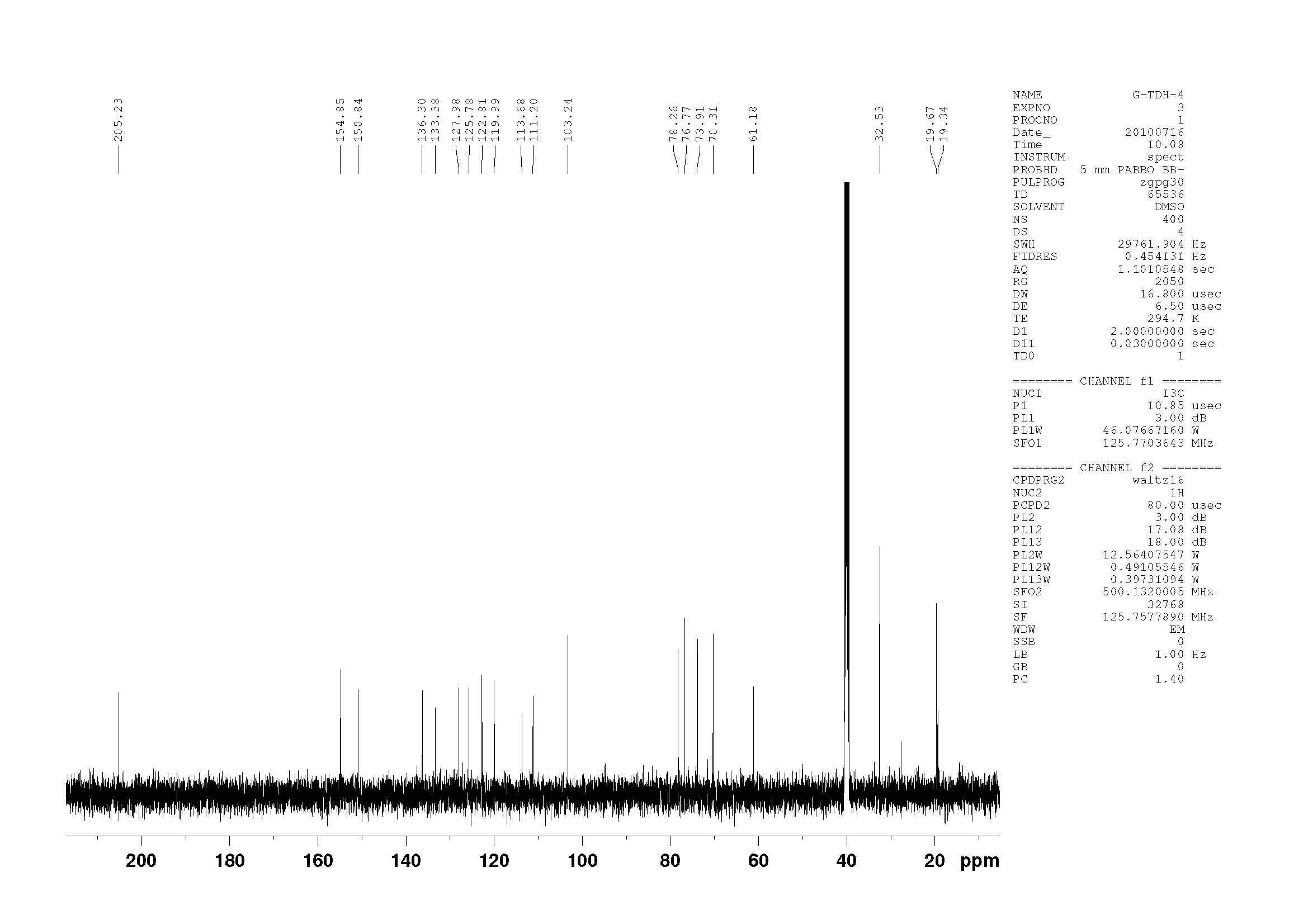


**Figure 16.** DEPT 135 spectrum of compound **3**.


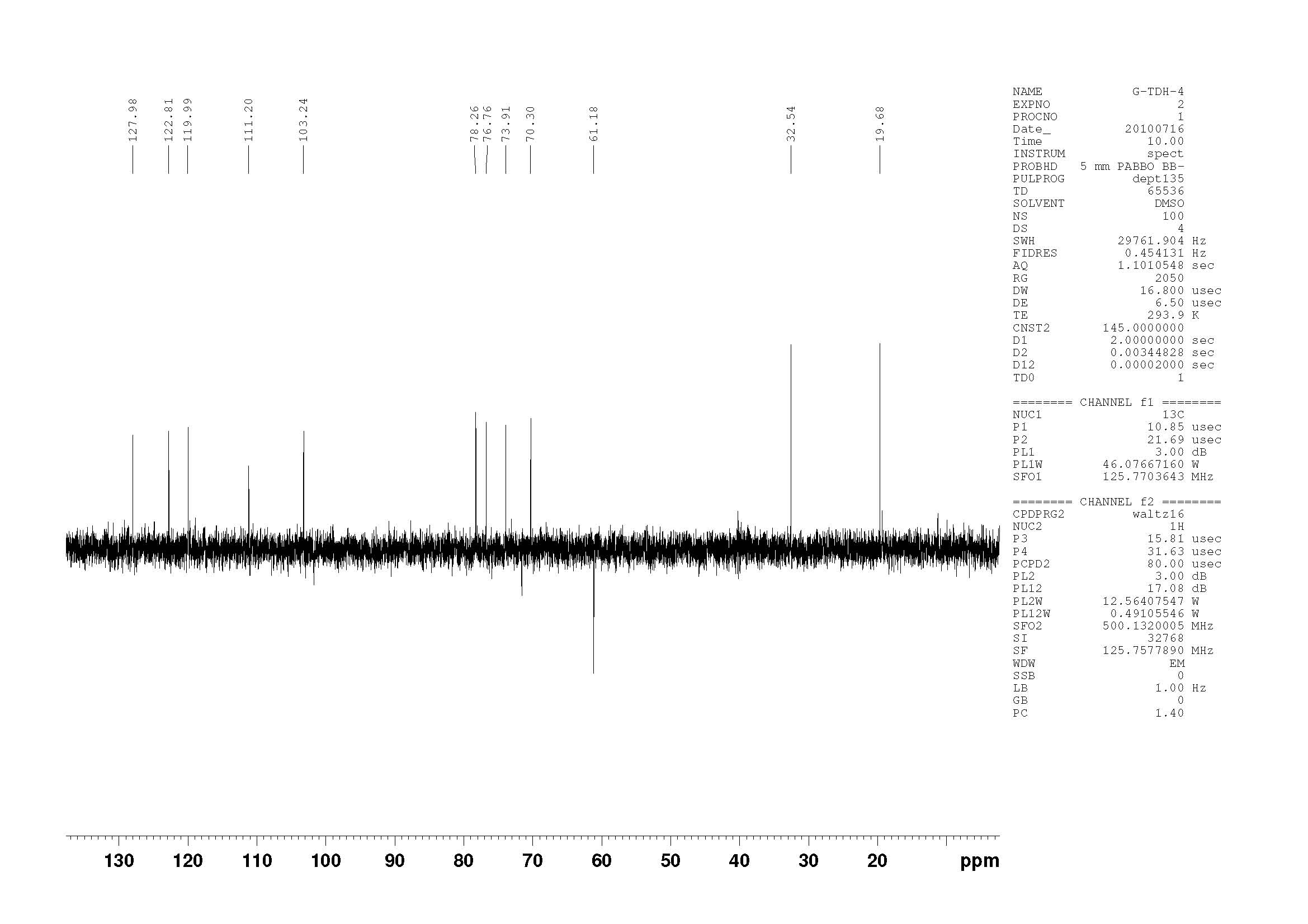


**Figure 17.** HSQC spectrum of compound **3**.


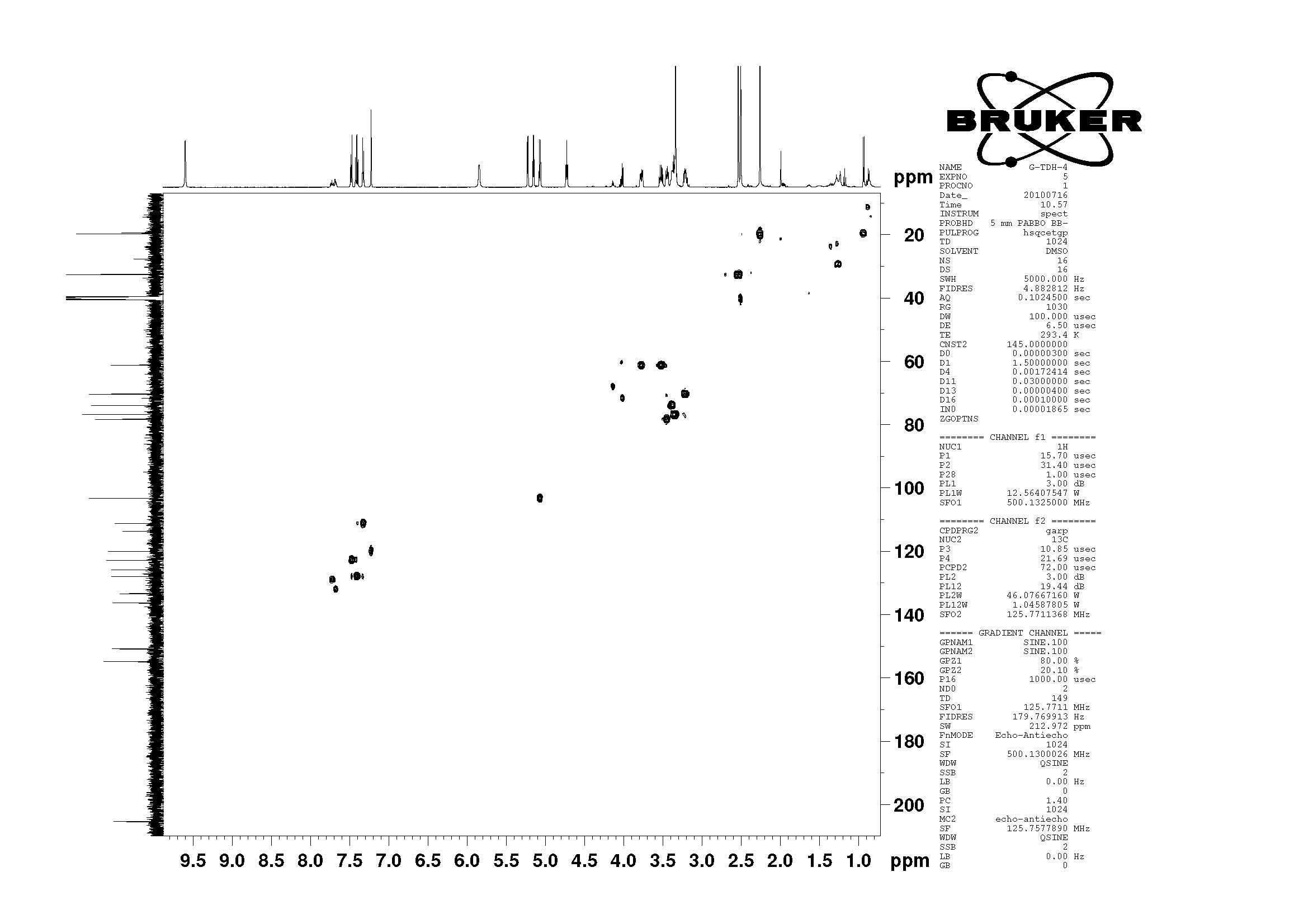


**Figure 18.** HSQC spectrum of compound **3**.


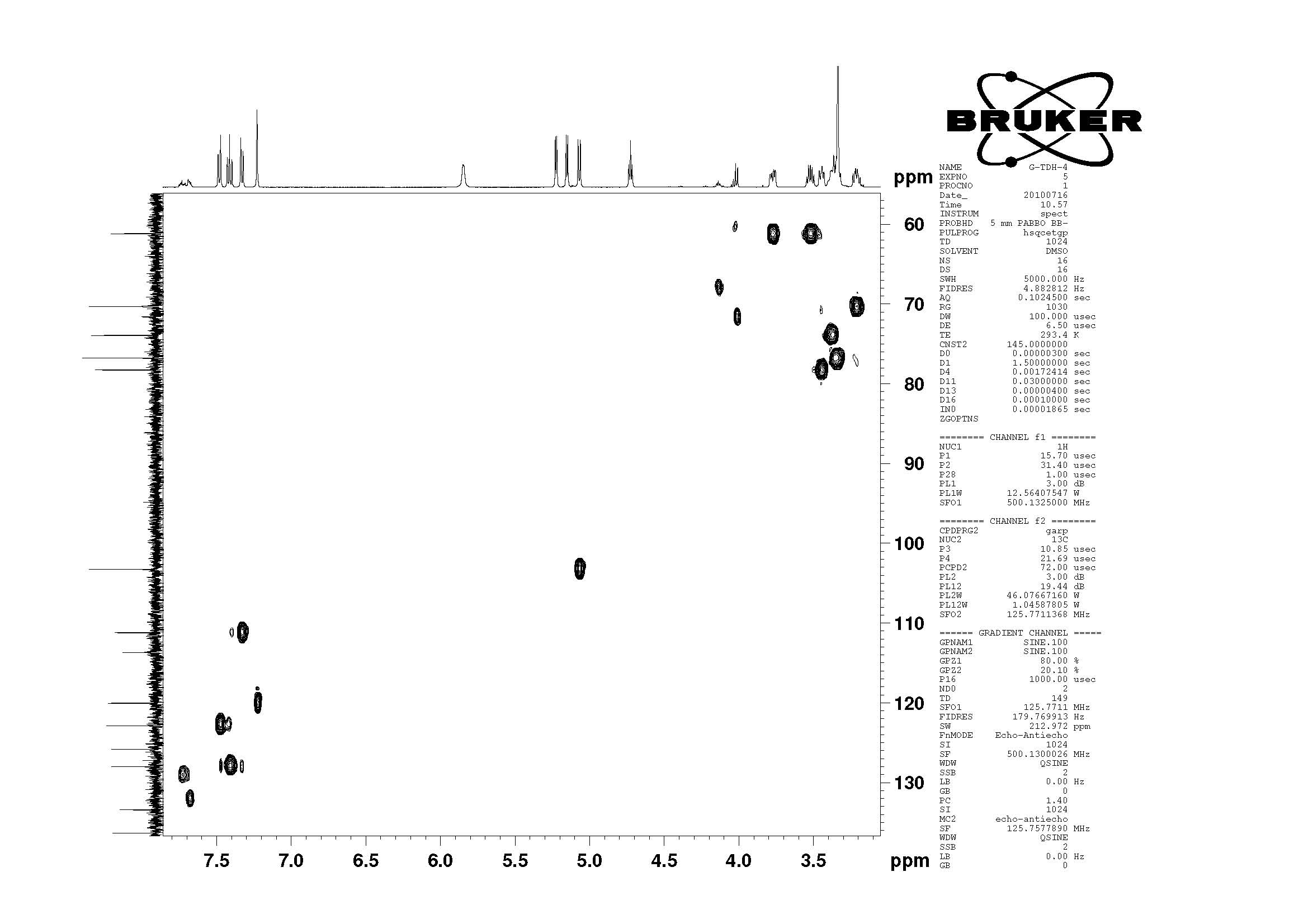


**Figure 19.** HMBC spectrum of compound **3**.


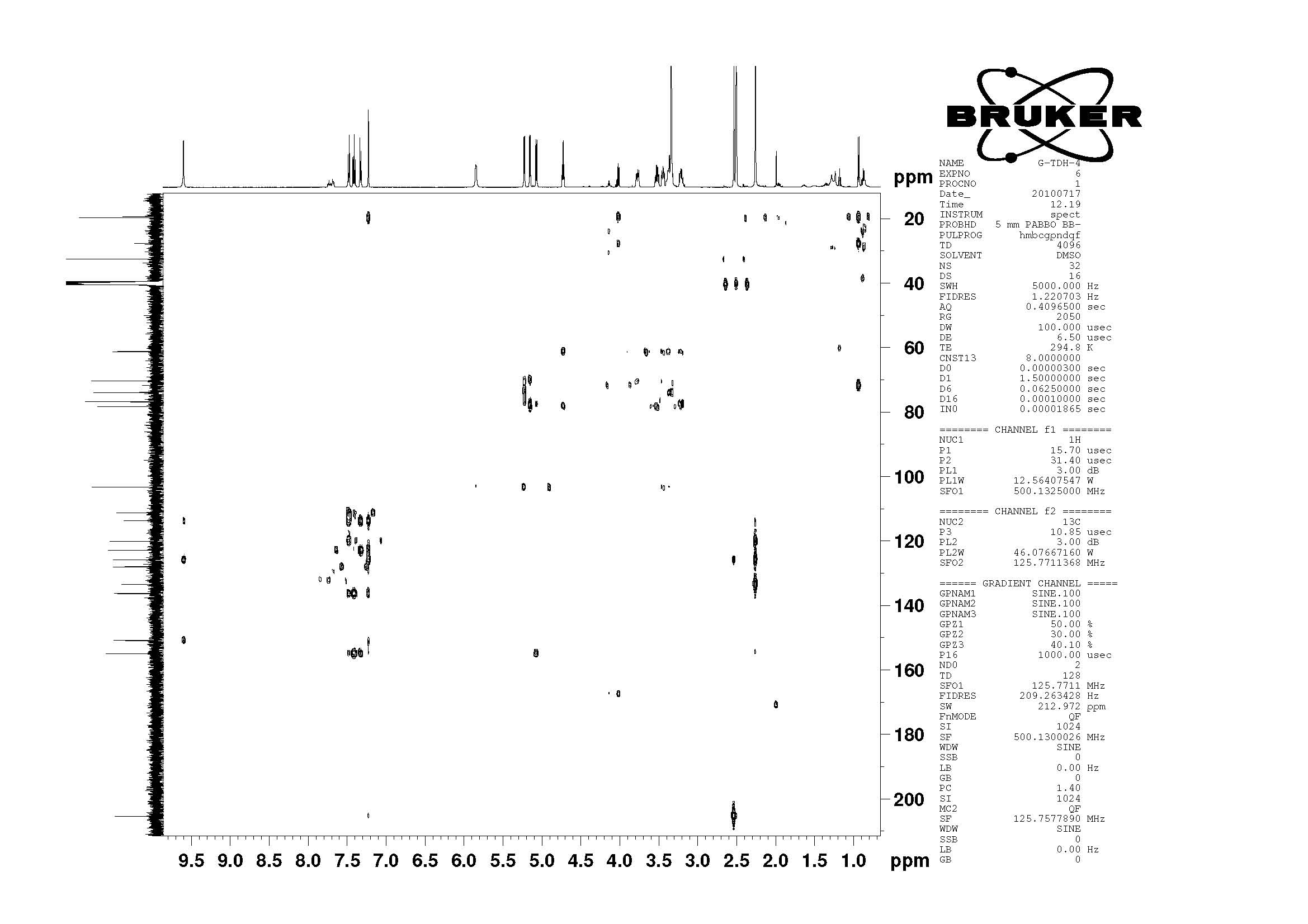


**Figure 20.** HMBC spectrum of compound **3**.


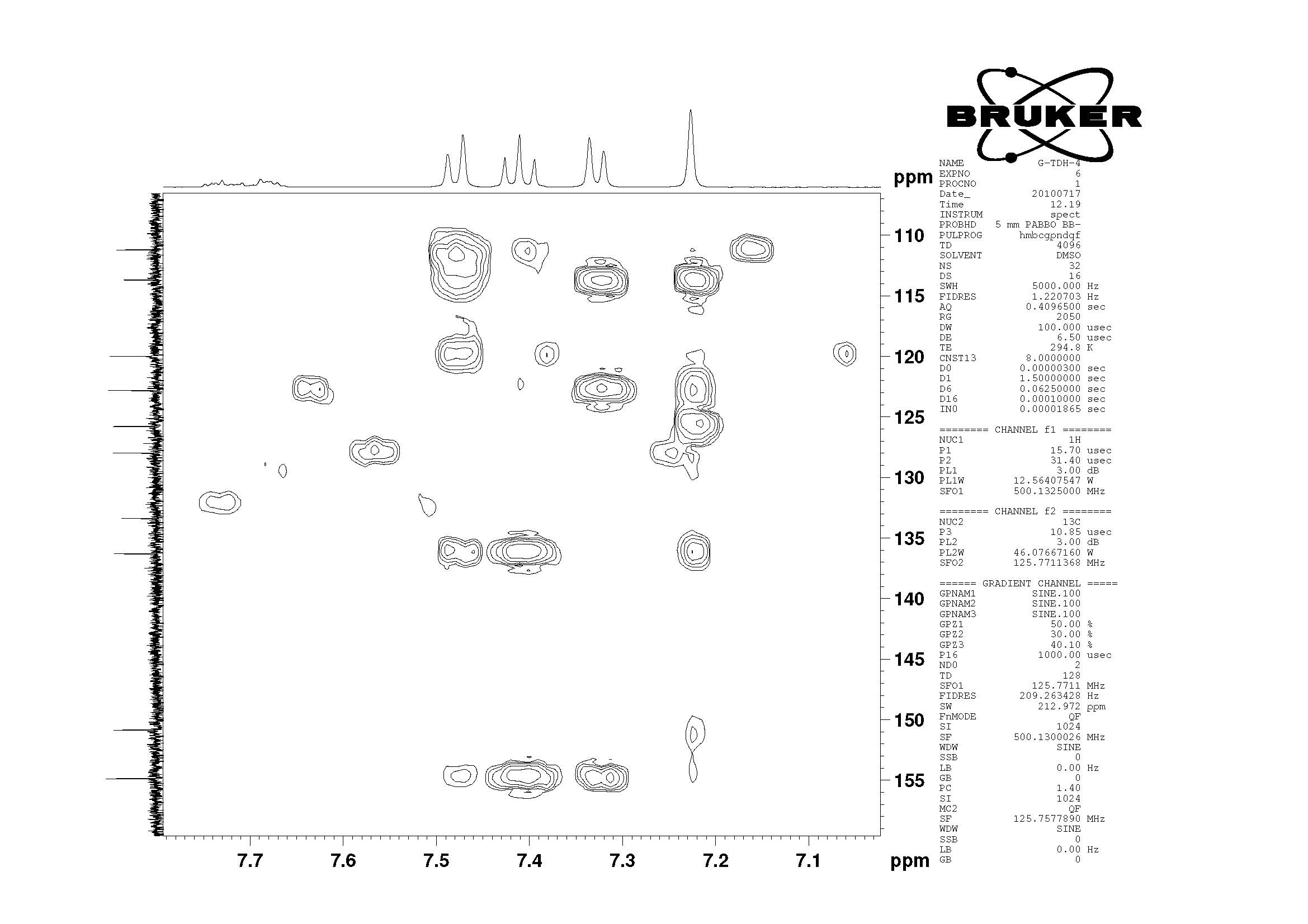


**Figure 21.** HMBC spectrum of compound **3**.


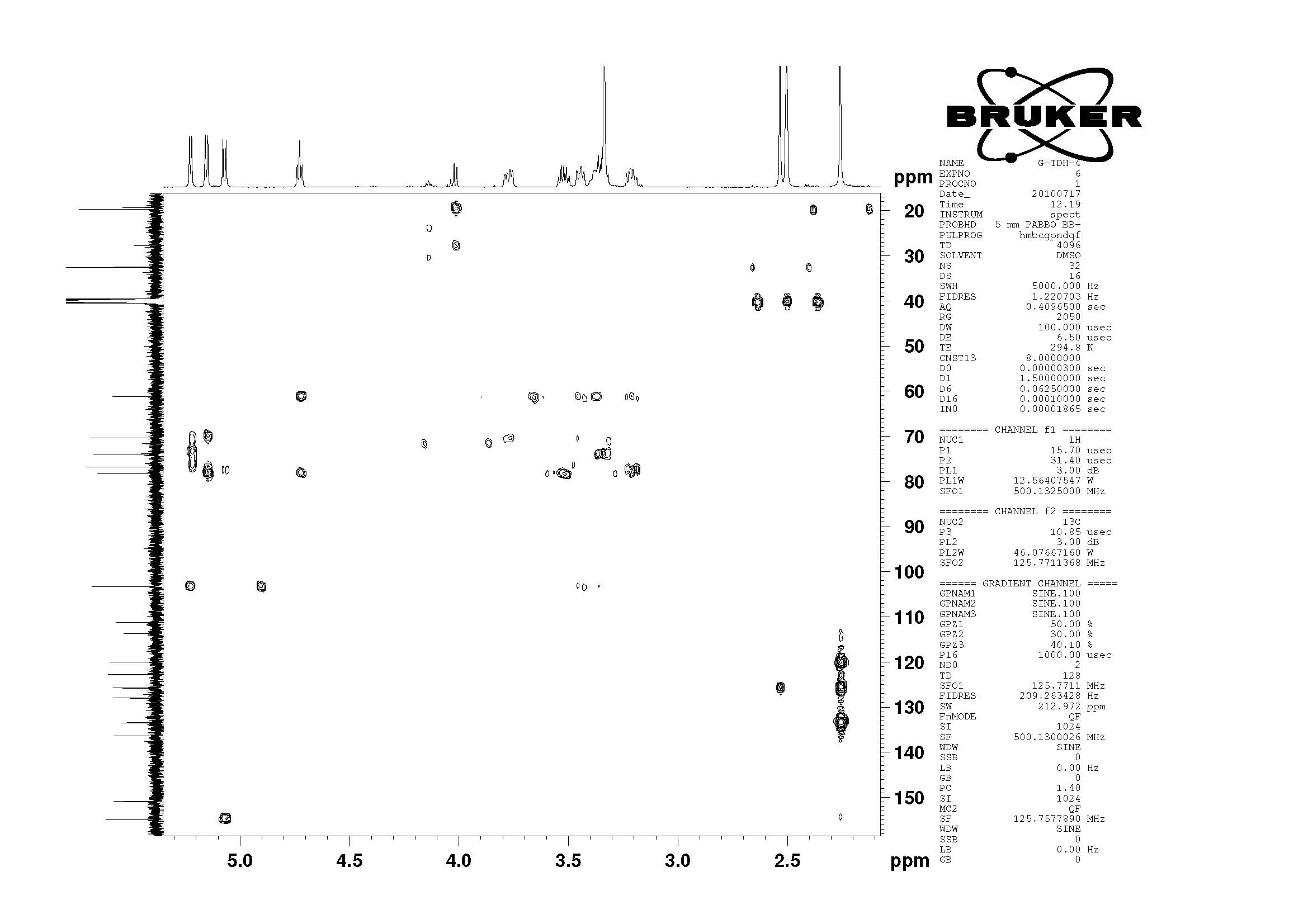


HPLC system: Waters 600 (Waters, USA)

Detector: PDA 996 (Waters, USA)

Chromatographic work station: Millennium^32^ (Waters, USA)

Column: Intersel C18 (5 μm, 4.6 × 250mm)

Mobile Phase: 40% MeOH/H_2_O

Wave length: 225 nm

**Figure 22.** HPLC chromatogram of compound **1**.


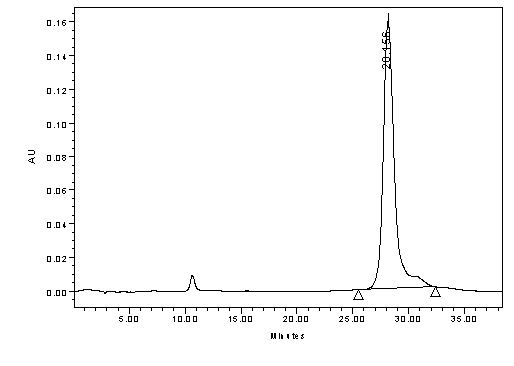


**Figure 23.** HPLC chromatogram of compound **2**.


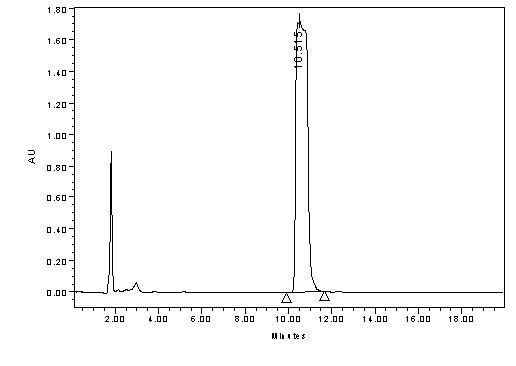


**Figure 24.** HPLC chromatogram of compound **3**.


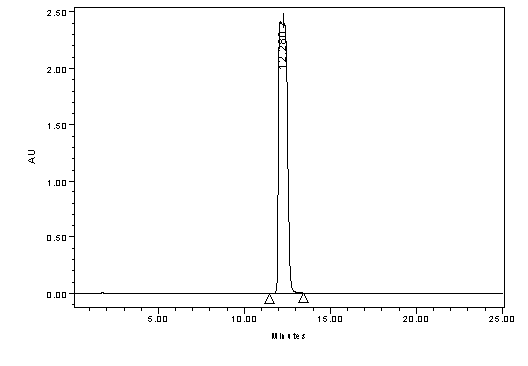


**Figure 25.** GC chromatogram of standard D-glucose.

**Figure 26.** GC chromatogram of the sugar after acid hydrolysis of compound **2**.


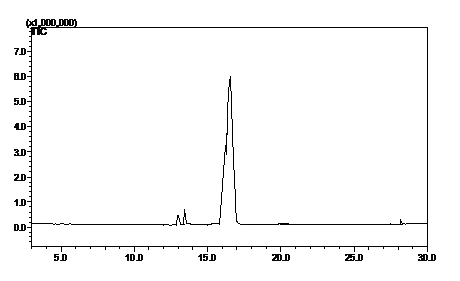


Inject Temp.: 280 °C

Column Temp.: 180 °C

Flow Rate: 1.0 mL/min

split ratio: 5:1

Heating Program: 180 °C for 10 min, increase to 280 °C at the speed of 10 °C/min, keep for 5 min

Column：HP-1
